# Comprehensive safety profile evaluation of bivalirudin in Chinese ST-segment elevation myocardial infarction patients receiving percutaneous coronary intervention: a prospective, multicenter, intensive monitoring study

**DOI:** 10.1186/s12872-022-02716-4

**Published:** 2022-06-25

**Authors:** Haijun Zheng, Zhonghua Wang, Qi Li, Yingxin Zhao, Yin Liu, Aiming Chen, Jianping Deng, Guohai Su

**Affiliations:** 1 Department of Cardiology, Jiaozuo People’s Hospital, Jiaozuo, China; 2 Department of Cardiology, Chenzhou First People’s Hospital, Chenzhou, China; 3 Department of Cardiology, Peking University People’s Hospital, Beijing, China; 4 Department of Cardiology, Beijing Institute of Heart Lung and Blood Vessel Disease, Beijing Anzhen Hospital, Capital Medical University, Beijing, China; 5 Department of Cardiology, Tianjin Chest Hospital, Tianjin, China; 6 Department of Cardiology, The First People’s Hospital of Jinzhou District, Dalian, China; 7 Department of Cardiology, Nanchong Central Hospital, No. 97, Renmin South Road, Shunqing District, Nanchong, 637000 China; 8 Department of Cardiology, Jinan Clinical Medical College, Jinan Central Hospital, Shan-Dong University, No. 105 Jiefang Road, Jinan, 250000 Jinan China

**Keywords:** Bivalirudin, Percutaneous coronary intervention, ST-segment elevation myocardial infarction, Adverse events and drug reactions, Thrombocytopenia and bleeding

## Abstract

**Background:**

This prospective, multi-center, intensive monitoring study aimed to systematically assess the occurrence of adverse events (AEs) and adverse drug reactions (ADRs), especially thrombocytopenia and bleeding, as well as their risk factors in Chinese ST-segment elevation myocardial infraction (STEMI) patients receiving bivalirudin as anticoagulant for percutaneous coronary intervention (PCI).

**Methods:**

In total, 1244 STEMI patients undergoing PCI and receiving bivalirudin as anticoagulant were enrolled in the present study. Safety data were collected from hospital admission to 72 h after bivalirudin administration; in addition, patients were further followed up at the 30th day with safety data collected at that time.

**Results:**

AEs, severe AEs, ADRs and severe ADRs were reported in 224 (18.0%), 15 (1.2%), 49 (3.9%) and 5 (0.4%) patients, respectively. Importantly, 4 (0.3%) patients were submitted to hospitalization and 6 (0.5%) patients died due to AEs, while 1 (0.1%) patient was submitted to hospitalization but no (0.0%) patient died due to ADRs. Meanwhile, thrombocytopenia and bleeding occurred in 24 (1.9%) and 21 (1.7%) patients, respectively. Further multivariate logistic analysis identified several important independent factors related to AEs, ADRs, thrombocytopenia or bleeding, which included history of cardiac surgery and renal function impairment, high CRUSADE risk stratification, elective operation and combination with glycoprotein IIb/IIIa inhibitors. Moreover, 4 multivariate models were constructed based on the above-mentioned factors, which all showed acceptable predictive value for AEs, ADRs, thrombocytopenia and bleeding, respectively.

**Conclusion:**

Bivalirudin is a well-tolerant anticoagulant in Chinese STEMI patients undergoing PCI procedure.

**Supplementary Information:**

The online version contains supplementary material available at 10.1186/s12872-022-02716-4.

## Background

Acute myocardial infarction (AMI) is one of the leading causes of morbidity and mortality worldwide [1]. In China, AMI induced mortality is sharply increasing during the past few decades; meanwhile, it is estimated that patients with AMI would be 23 million by 2030 [2, 3]. Considering that the risk factors of AMI including aging, obesity and smoking are becoming more prevalent in China, AMI would be a heavy burden on the public health system [4–6]. ST-segment elevation myocardial infarction (STEMI) is the main manifestation of AMI, which is considered the top reason for premature death all over the world [7, 8]. Fortunately, percutaneous coronary intervention (PCI) is an effective management that greatly reduces the mortality of patients with STEMI [9, 10]. At present, anticoagulants such as unfractionated heparin with or without glycoprotein (GP) IIb/IIIa inhibitors are widely administrated to patients undergoing PCI because they greatly reduce the risk of PCI-related complications such as in-stent thrombosis [11]. On the other hand, anticoagulants might also induce several adverse events including bleeding and thrombocytopenia, which inversely increase the mortality risk [12, 13].

Bivalirudin is an oligopeptide that directly inhibits thrombin, thus being considered as a potential anticoagulant [14]. Although the efficiency of bivalirudin over heparin is controversial, several large-scale trials have shown that bivalirudin ameliorates complications associated with anticoagulants including bleeding compared to heparin with or without GP IIb/IIIa inhibitors in STEMI patients receiving PCI [15–21]. However, due to the fact that bivalirudin is recently applied in China, there lacks large-scale analysis to investigate the safety of bivalirudin in Chinese STEMI patients receiving PCI, which restricts its clinical application.

The present study prospectively enrolled 1244 STEMI patients from 27 centers in China, aimed to intensively monitor the occurrences of adverse events (AEs), adverse drug reactions (ADRs), especially thrombocytopenia as well as bleeding, and to investigate their risk factors in Chinese STEMI patients taking bivalirudin as anticoagulant for PCI.

## Methods

### Patients

We performed a prespecified subgroup analysis of 1244 STEMI patients from a prospective, multi-center, intensive monitoring study which enrolled 3049 patients who underwent PCI and received bivalirudin as anticoagulant in 27 Chinese medical centers and aimed to evaluate the safety of bivalirudin in a wide range of population. These 1244 patients were chosen based on the criterium of having STEMI. During the process of the study, the therapy of all patients and the medication use were determined by attending physicians on the basis of the actual clinical situation, and were not interfered by the study. This study was approved by the Ethics Committee of each participant center (Jiaozuo People's Hospital; Chenzhou First People's Hospital; Peking University People's Hospital; Beijing Institute of Heart Lung and Blood Vessel Disease; Beijing Anzhen Hospital, Capital Medical University; Tianjin Chest Hospital; The First People's Hospital of Jinzhou District, Nanchong Central Hospital; and Jinan Clinical Medical College, Jinan Central Hospital, Shan-dong University). The trial was conducted in accordance with the Declaration of Helsinki, and he written informed consents were obtained from all study participants.

### Collection of clinical data

The following clinical data were collected: (1) demographic characteristics: age, gender and body mass indexes (BMI); (2) medial history: history of diabetes mellitus, history of critical respiratory disease, history of renal function impairment, history of allergy and history of cardiac surgery; (3) CRUSADE score (Can Rapid Risk Stratification of Unstable Angina Patients Suppress Adverse Outcomes with Early Implementation of the ACC/AHA Guidelines- bleeding score [22]); (4) PCI characteristics: operative timing, types of coronary interventional therapy, types of stents, arterial access and culprit vessel; (5) administration of bivalirudin: preoperative or intraoperative, postoperative ≤ 4 h and postoperative > 4 h; (6) combined with GP IIb/IIIa inhibitors.

### Collection of safety data

Safety data were collected from hospital admission to 72 h after completion of bivalirudin administration, in addition, patients were further followed up at the 30th day, and the safety data were also collected at that time. AEs and ADRs were classified using the Systematic Organ Classification (SOC) and Preferred Term (PT) of the International Conference on the Coordination of International Drug Registration (ICH) Medical Dictionary for Regulatory Activities (MedDRA) 23.0.

### Definitions

AEs were defined as any unfavorable and unintended sign (including an abnormal laboratory finding), symptom, or disease temporally associated with the use of a medical treatment that may or may not be considered related to the medical treatment. ADRs were defined as the harmful reaction of qualified drugs which was irrelevant to the purpose of medication under normal usage and dosage. Severe adverse events (SAEs) and severe adverse drug reactions (SADRs) were defined as one of the following events: (1) leading to death; (2) life-threatening consequences; (3) leading to carcinogenesis, teratogenesis and birth defects; (4) leading to significant or permanent human disability or organ function damage; (5) resulting in hospitalization or prolonged length of stay; (6) leading to other important medical events, if not treated, the above listed conditions may occur. Severity of AEs and ADRs was divided into three levels according to the following criteria: (1) mild: symptoms were transient and did not affect the patient's normal daily activities; (2) moderate: symptoms were obvious to affect the patient's normal daily activities, but tolerable, which were not required to stop medication; (3) severe: symptoms were obvious, intolerable and affected the patient's normal daily activities, which were required to stop medication.

### Statistical analysis

Descriptive analyses were performed using mean value ± standard deviation (SD) or count (percentage) as appropriate. Univariate analyses were performed using Chi-square test or Fisher's exact test. Predictive factors of AEs, ADRs, thrombocytopenia and bleeding were analyzed by logistic regression model with all factors listed in Table 1 (including demographic characteristics, medical history, CRUSADE score, PCI characteristics, administration time of bivalirudin and combination with GP IIb/III a inhibitors) and screened using forward method. Then, the performance of the multivariate model was estimated using receiver operating characteristic (ROC) curve analysis. A *P* < 0.05 was considered statistically significant. SAS 9.4 (SAS Institute, Inc., Cary, North Carolina, USA) was applied to complete data analysis.

**Table 1:** Clinical characteristics

Items	STEMI patients (N = 1244)
*Demographic characteristics*	
Age (years), mean ± SD	63.9 ± 11.9
Male, No. (%)	915 (73.6)
BMI (kg/m^2^), mean ± SD	25.3 ± 31.4
*Medical history*	
History of diabetes mellitus, No. (%)	251 (20.2)
History of allergy, No. (%)	87 (7.0)
History of cardiac surgery, No. (%)	62 (5.0)
History of critical respiratory disease, No. (%)	36 (2.9)
History of renal function impairment, No. (%)	22 (1.8)
*CRUSADE score*	
Mean ± SD	30.2 ± 14.5
Risk stratification, No. (%)	
Very low risk (≤ 20)	328 (26.4)
Low risk (21–30)	350 (28.1)
Moderate risk (31–40)	277 (22.3)
High risk (41–50)	157 (12.6)
Very high risk (> 50)	113 (9.1)
Unknown	19 (1.5)
*PCI characteristics*	
Operative timing, No. (%)	
Emergency operation	938 (75.4)
Elective operation	306 (24.6)
Types of coronary interventional therapy, No. (%)	
Stent implantation	1184 (95.2)
Balloon dilatation	56 (4.5)
Thrombus aspiration	0 (0.0)
Others	4 (0.3)
Types of stents, No. (%)	
Drug stent	1143 (91.9)
Bare stent	42 (3.4)
Others	1 (0.1)
Unknown	58 (4.7)
Arterial access, No. (%)	
Radial artery	1180 (94.9)
Femoral artery	61 (4.9)
Brachial artery	0 (0.0)
Others	3 (0.2)
Culprit vessel, No. (%)	
Single	1053 (84.6)
Multiple	190 (15.3)
Unknown	1 (0.1)
*Administration of bivalirudin*	
Preoperative or intraoperative, No. (%)	44 (3.5)
Postoperative ≤ 4 h, No. (%)	1116 (89.7)
Postoperative > 4 h, No. (%)	84 (6.8)
*Combined with GP IIb/IIIa inhibitors, No. (%)*	805 (64.7)

STEMI, ST-segment elevation myocardial infarction; SD, standard deviation; BMI, body mass indexes; CRUSADE, Can Rapid Risk Stratification of Unstable Angina Patients Suppress Adverse Outcomes with Early Implementation of the ACC/AHA Guidelines; PCI, percutaneous coronary intervention; GP, glycoprotein

## Results

### Characteristics of STEMI patients

The STEMI patients had a mean age of 63.9 ± 11.9 years with 915 (73.6%) males; meanwhile, the mean CRUSADE score was 30.2 ± 14.5. Regarding the PCI detail, 938 (75.4%) patients received emergency operation while 306 (24.6%) patients received elective operation. Besides, 1184 (95.2%), 56 (4.5%), 0 (0.0%), and 4 (0.3%) patients experienced stent implantation, balloon dilatation, thrombus aspiration and other coronary interventional therapies, respectively; 1143 (91.9%), 42 (3.4%), 1 (0.1%) and 58 (4.7%) patients received drug stent, bare stent, other stents or unknown stents, accordingly. In addition, the numbers of patients received PCI through radial artery, femoral artery, brachial artery and other accesses were 1180 (94.9%), 61 (4.9%), 0 (0.0%) and 3 (0.2%), respectively. With respect to the administration time of bivalirudin, 44 (3.5%) patients were given preoperative or intraoperative bivalirudin, 1116 (89.7%) patients received bivalirudin within 4 h of PCI, and 84 (6.8%) patients were administrated with bivalirudin after 4 h of PCI. Moreover, 805 (64.7%) patients received bivalirudin combined with GP IIb/IIIa inhibitors. The detailed clinical and PCI characteristics of STEMI patients were shown in Table 1.

### Incidence of AEs, ADRs, thrombocytopenia and bleeding

**Fig. 1:**
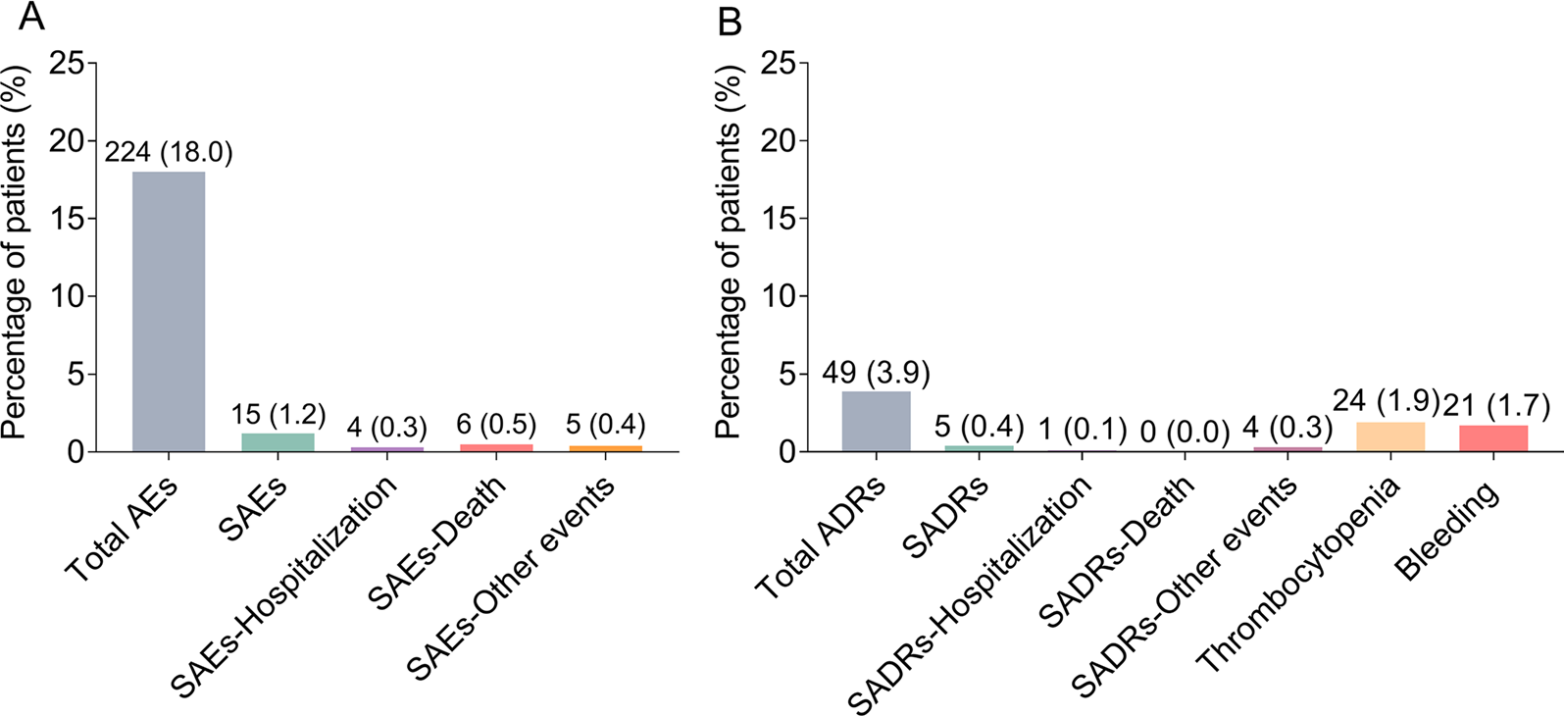
Incidence of AEs, ADRs, thrombocytopenia and bleeding. Description of AEs (**A**), ADRs, thrombocytopenia and bleeding (**B**) in Chinese STEMI patients receiving PCI and bivalirudin as anticoagulant. AEs: adverse events; ADRs: adverse drug reactions; STEMI: ST-segment elevation myocardial infarction; PCI: percutaneous coronary intervention

A total of 224 (18.0%) patients experienced AEs. Meanwhile, SAEs were reported in 15 (1.2%) patients. Additionally, the SAEs resulted in 4 (0.3%) cases of hospitalization, 6 (0.5%) cases of mortality and 5 (0.4%) cases of other events (Fig. 1A).

Concerning ADRs, they were reported in 49 (3.9%) patients, among which 5 (0.4%) patients suffered from SADRs. In addition, 1 (0.1%) patient was admitted to hospitalization due to SADRs, no (0.0%) patient died from SADRs, and 4 (0.3%) patients experienced other events due to SADRs. Notably, thrombocytopenia and bleeding occurred in 24 (1.9%) and 21 (1.7%) patients, respectively (Fig. 1B).

The specific AEs and ADRs in SOC were presented in Additional file 1: Table 1, which showed that most of the AEs and ADRs were mild to moderate.

### AEs, ADRs, thrombocytopenia and bleeding stratified by baseline criteria

Age > 75 years (*P* = 0.041), history of cardiac surgery (*P* = 0.008), critical respiratory diseases (*P* < 0.001) and renal function impairment (*P* = 0.043), emergency operation (*P* < 0.001), as well as administration of bivalirudin combined with GP IIb/IIIa inhibitors (*P* < 0.001) were correlated with higher incidence of AEs. Meanwhile, history of cardiac surgery (*P* = 0.009) and renal function impairment (*P* = 0.009), higher CRUSADE risk stratification (*P* < 0.001), as well as administration of bivalirudin combined with GP IIb/IIIa inhibitors (*P* = 0.002) were correlated with elevated incidence of ADRs. Apart from that, history of cardiac surgery (*P* = 0.005), higher CRUSADE risk stratification (*P* = 0.009), elective operation (*P* = 0.004) and administration of bivalirudin combined with GP IIb/IIIa inhibitors (*P* = 0.005) were correlated with increased incidence of thrombocytopenia. Besides, higher CRUSADE risk stratification (*P* = 0.011) and emergency operation (*P* = 0.033) were correlated with higher risk of bleeding (Table 2).

**Table 2:** AEs, ADRs, thrombocytopenia and bleeding stratified by baseline criteria

Items	AEs	*P* value	ADRs	*P* value	Thrombocytopenia	*P* value	Bleeding	*P* value
Age, no. (%)		**0.041**		0.168		0.282		0.236
> 75 years	49 (22.9)		12 (5.6)		6 (2.8)		6 (2.8)	
≤ 75 years	175 (17.0)		37 (3.6)		18 (1.7)		15 (1.5)	
Gender, No. (%)		0.968		0.182		0.088		0.471
Male	165 (18.0)		32 (3.5)		14 (1.5)		14 (1.5)	
Female	59 (17.9)		17 (5.2)		10 (3.0)		7 (2.1)	
BMI, No. (%)		0.776		0.424		0.254		1.000
> 28 kg/m^2^	20 (19.8)		2 (2.0)		0 (0.0)		1 (1.0)	
≤ 28 kg/m^2^	195 (18.6)		44 (4.2)		23 (2.2)		18 (1.7)	
History of diabetes mellitus, No. (%)		0.071		0.135		0.301		0.407
Yes	55 (21.9)		14 (5.6)		7 (2.8)		6 (2.4)	
No	169 (17.0)		35 (3.5)		17 (1.7)		15 (1.5)	
History of allergy, No. (%)		0.847		0.772		0.682		0.654
Yes	15 (17.2)		4 (4.6)		2 (2.3)		2 (2.3)	
No	209 (18.1)		45 (3.9)		22 (1.9)		19 (1.6)	
History of cardiac surgery, No. (%)		**0.008**		**0.009**		**0.005**		0.282
Yes	19 (30.6)		7 (11.3)		5 (8.1)		2 (3.2)	
No	205 (17.3)		42 (3.6)		19 (1.6)		19 (1.6)	
History of critical respiratory disease, No. (%)		** < 0.001**		0.650		1.000		0.463
Yes	16 (44.4)		2 (5.6)		0 (0.0)		1 (2.8)	
No	208 (17.2)		47 (3.9)		24 (2.0)		20 (1.7)	
History of renal function impairment, No. (%)		**0.043**		**0.009**		0.065		0.051
Yes	8 (36.4)		4 (18.2)		2 (9.1)		2 (9.1)	
No	216 (17.7)		45 (3.7)		22 (1.8)		19 (1.6)	
CRUSADE risk stratification, No. (%)		0.075		** < 0.001**		**0.009**		**0.011**
Very low risk (≤ 20)	55 (16.8)		11 (3.4)		3 (0.9)		4 (1.2)	
Low risk (21–30)	56 (16.0)		3 (0.9)		2 (0.6)		1 (0.3)	
Moderate risk (31–40)	53 (19.1)		17 (6.1)		11 (4.0)		6 (2.2)	
High risk (41–50)	24 (15.3)		7 (4.5)		3 (1.9)		4 (2.5)	
Very high risk (> 50)	31 (27.4)		11 (9.7)		5 (4.4)		6 (5.3)	
Unknown	5 (26.3)		0 (0.0)		0 (0.0)		0 (0.0)	
Operative timing, No. (%)		** < 0.001**		0.749		**0.004**		**0.033**
Elective operation	31 (10.1)		13 (4.2)		12 (3.9)		1 (0.3)	
Emergency operation	193 (20.6)		36 (3.8)		12 (1.3)		20 (2.1)	
Types of coronary interventional therapy, No. (%)		0.101		0.640		0.958		0.965
Stent implantation	207 (17.5)		48 (4.1)		23 (1.9)		20 (1.7)	
Balloon dilatation	16 (28.6)		1 (1.8)		1 (1.8)		1 (1.8)	
Thrombus aspiration	0 (0.0)		0 (0.0)		0 (0.0)		0 (0.0)	
Others	1 (25.0)		0 (0.0)		0 (0.0)		0 (0.0)	
Types of stents, No. (%)		0.564		0.837		0.643		0.931
Drug stent	202 (17.7)		47 (4.1)		23 (2.0)		19 (1.7)	
Bare stent	5 (11.9)		1 (2.4)		0 (0.0)		1 (2.4)	
Others	0 (0.0)		0 (0.0)		0 (0.0)		0 (0.0)	
Arterial access, No. (%)		0.568		0.868		0.715		0.974
Radial artery	215 (18.2)		46 (3.9)		22 (1.9)		20 (1.7)	
Femoral artery	9 (14.8)		3 (4.9)		2 (3.3)		1 (1.6)	
Brachial artery	0 (0.0)		0 (0.0)		0 (0.0		0 (0.0)	
Others	0 (0.0)		0 (0.0)		0 (0.0)		0 (0.0)	
Culprit vessel, No. (%)		0.224		0.836		0.777		0.548
Single	183 (17.4)		41 (3.9)		20 (1.9)		17 (1.6)	
Multiple	40 (21.1)		8 (4.2)		4 (2.1)		4 (2.1)	
Administration of bivalirudin, No. (%)		0.112		0.355		0.408		0.830
Preoperative or intraoperative	9 (20.5)		3 (6.8)		2 (4.5)		1 (2.3)	
Postoperative ≤ 4 h	193 (17.3)		41 (3.7)		20 (1.8)		18 (1.6)	
Postoperative > 4 h	22 (26.2)		5 (6.0)		2 (2.4)		2 (2.4)	
Combined with GP IIb/IIIa inhibitors, no. (%)		** < 0.001**		**0.002**		**0.005**		0.267
Yes	192 (23.9)		42 (5.2)		22 (2.7)		16 (2.0)	
No	32 (7.3)		7 (1.6)		2 (0.5)		5 (1.1)	

The bold indicates items of statistical significance

AEs, adverse events; ADRs, adverse drug reactions; BMI, body mass indexes; CRUSADE, Can Rapid Risk Stratification of Unstable Angina Patients Suppress Adverse Outcomes with Early Implementation of the ACC/AHA Guidelines; GP, glycoprotein

### Independent factors for AEs, ADRs, thrombocytopenia and bleeding

**Fig. 2:**
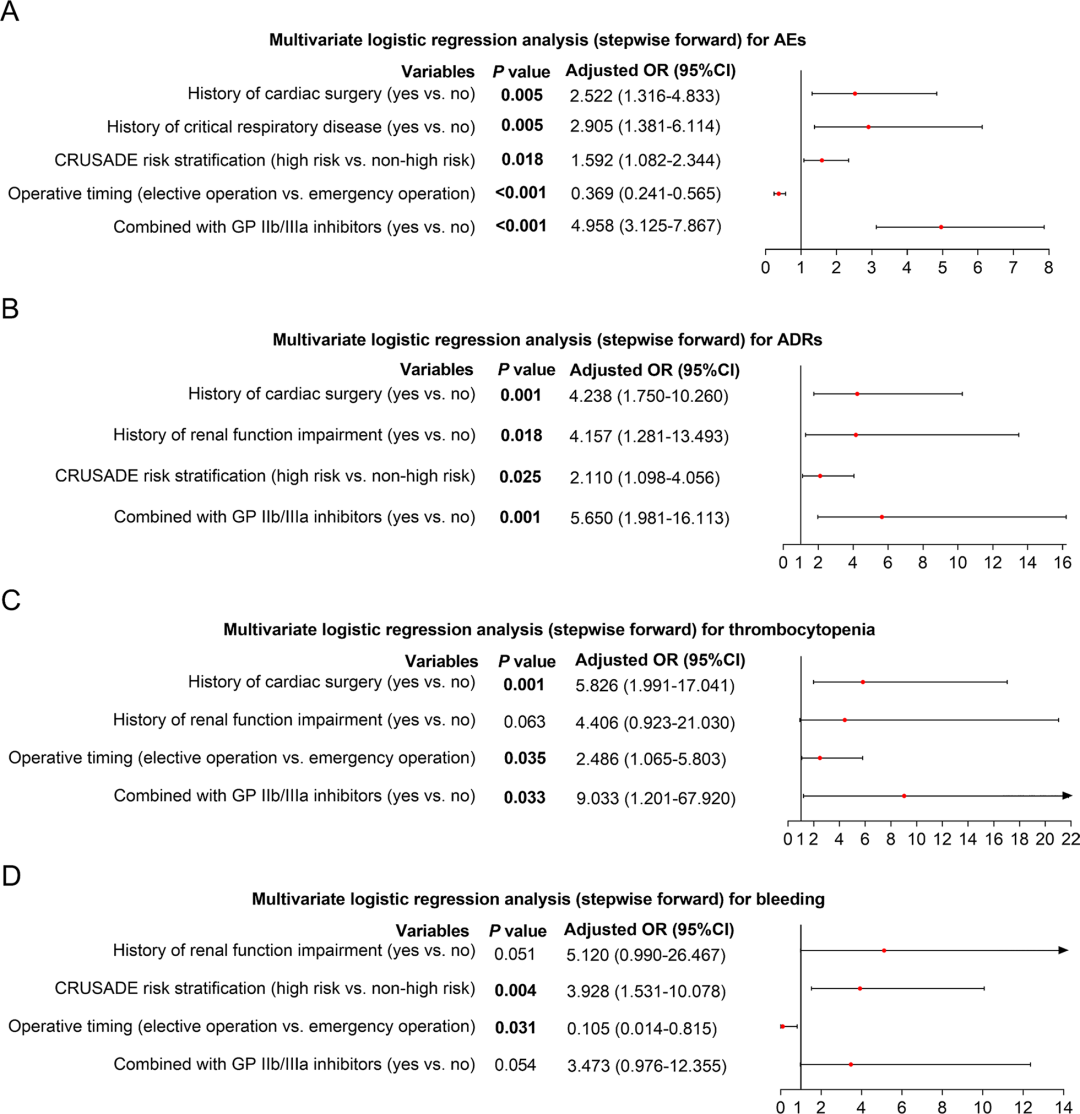
Multivariate logistic regression analysis. Independent risk factors for AEs (**A**), ADRs (**B**), thrombocytopenia (**C**) and bleeding (**D**) in Chinese STEMI patients receiving PCI and bivalirudin as anticoagulant. AEs: adverse events; ADRs: adverse drug reactions; STEMI: ST-segment elevation myocardial infarction; PCI: percutaneous coronary intervention

Multivariate logistic regression analyses were conducted to investigate the risk factors for AEs, ADRs, thrombocytopenia and bleeding. Data showed that history of cardiac surgery (*P* = 0.005, OR = 2.522) and critical respiratory disease (*P* = 0.005, OR = 2.905), high CRUSADE risk stratification (*P* = 0.018, OR = 1.592), and administration of bivalirudin combined with GP IIb/IIIa inhibitors (*P* < 0.001, OR = 4.958) were independent factors for higher incidence of AEs; while elective operation (*P* < 0.001, OR = 0.369) was an independent factor for lower incidence of AEs (Fig. 2A).

Regarding ADRs, history of cardiac surgery (*P* < 0.001, OR = 4.238) and renal function impairment (*P* = 0.018, OR = 4.157), high CRUSADE risk stratification (*P* = 0.025, OR = 2.110) and administration of bivalirudin combined with GP IIb/IIIa inhibitors (*P* = 0.001, OR = 5.650) were independently correlated with higher risk of ADRs (Fig. 2B).

Concerning thrombocytopenia, history of cardiac surgery (*P* = 0.001, OR = 5.826), elective operation (*P* = 0.035, OR = 2.486) and administration of bivalirudin combined with GP IIb/IIIa inhibitors (*P* = 0.033, OR = 9.033) were independent risk factors for thrombocytopenia. Meanwhile, history of renal function impairment (*P* = 0.063, OR = 4.406) was not an independent risk factor for thrombocytopenia (Fig. 2C).

With regard to bleeding, high CRUSADE risk stratification (*P* = 0.004, OR = 3.928) was independently associated with higher risk of bleeding, while elective operation (*P* = 0.031, OR = 0.105) was independently associated with lower risk of bleeding. However, history of renal function impairment (*P* = 0.051, OR = 5.120) and administration of bivalirudin combined with GP IIb/IIIa inhibitors (*P* = 0.054, OR = 3.473) were not independently correlated with risk of bleeding (Fig. 2D).

**Fig. 3:**
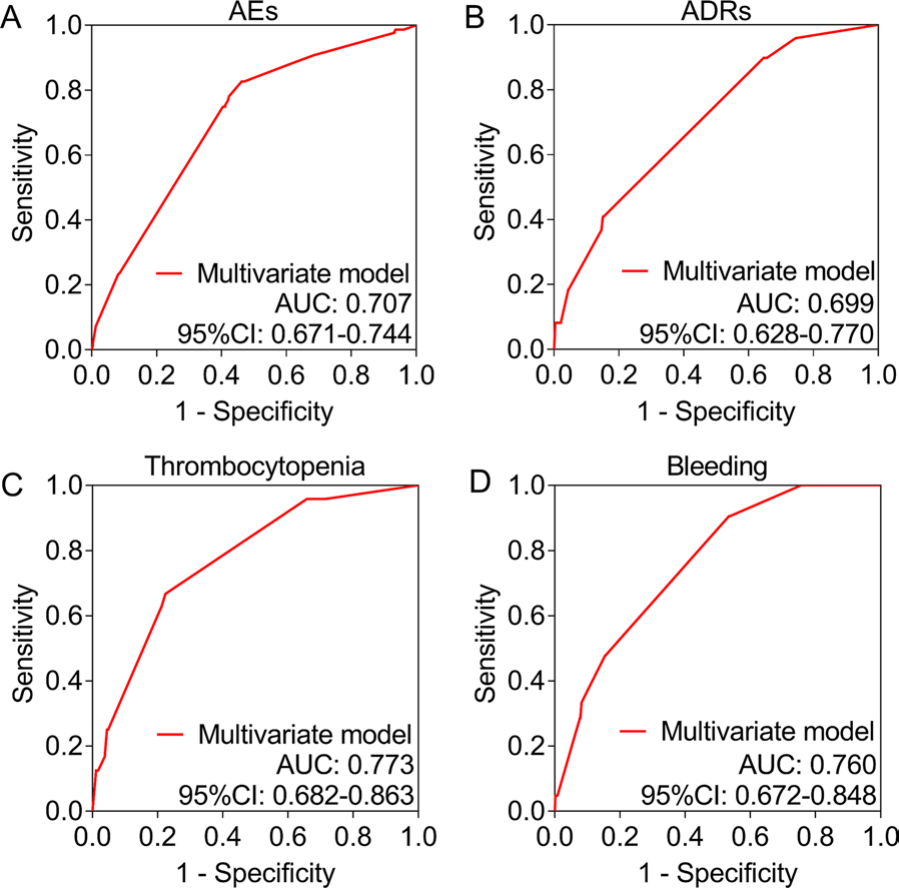
ROC curves. Predictive value of multivariate models for AEs (**A**), ADRs (**B**), thrombocytopenia (**C**) and bleeding (**D**) in Chinese STEMI patients receiving PCI and bivalirudin as anticoagulant. The ROC curve analysis was conducted based on the probability of the incidence to the events in each patient. The probability was calculated from the formulars generated by the multivariate logistic regression analysis. The formulars were listed as follows: $P\left(\mathrm{AEs}=\left.1\right|\mathrm{x}\right)=\frac{{e}^{g\left(x\right)}}{1+{e}^{g\left(x\right)}}$. $g\left(x\right)=-1.721+0.925\times \mathrm{History\, of\, cardiac\, surgery}+1.067\times \mathrm{History\, of \,critical\, respiratory\, disease}+0.465\times \mathrm{CRUSADE\, risk\, stratification}-0.997\times \mathrm{Operative \,timing}+1.601\times \mathrm{Combined \,with \,glycoprotein \,IIb}/\mathrm{IIIa \,inhibitors}$. $P\left(\mathrm{ADRs}=\left.1\right|\mathrm{x}\right)=\frac{{e}^{g\left(x\right)}}{1+{e}^{g\left(x\right)}}$. $g\left(x\right)=-4.902+1.444\times \mathrm{History \,of \,cardiac\, surgery}+1.425\times \mathrm{History\, of \,renal\, function \,impairment}+0.747\times \mathrm{CRUSADE\, risk\, stratification}+1.732\times \mathrm{Combined\, with \,glycoprotein \,IIb}/\mathrm{IIIa \,inhibitors}$. $P\left(\mathrm{thrombocytopenia}=\left.1\right|\mathrm{x}\right)=\frac{{e}^{g\left(x\right)}}{1+{e}^{g\left(x\right)}}$. $g(x)=-7.215+1.762\times \mathrm{History\, of \,cardiac\, surgery}+1.483\times \mathrm{History\, of\, renal\, function\, impairment}+0.911\times \mathrm{Operative\, timing}+2.201\times \mathrm{Combined \,with\, glycoprotein\, IIb}/\mathrm{IIIa \, inhibitors}$. ROC, receiver’s operating characteristics; AUC, area under curve; CI, confidence interval; AEs, adverse events; ADRs, adverse drug reactions; STEMI, ST-segment elevation myocardial infarction; PCI, percutaneous coronary intervention

Moreover, 4 multivariate model were constructed based on the above-mentioned factors to predict the risk of AEs, ADRs, thrombocytopenia and bleeding, respectively. Data showed that all the 4 multivariate model presented acceptable value in predicting AEs, ADRs, thrombocytopenia and bleeding, accordingly (Fig. 3A–D).

## Discussion

The findings of the present study highlighted that in Chinese STEMI patients receiving PCI and bivalirudin as anticoagulant, the incidences of AEs, SAEs, ADRs, SADRs, thrombocytopenia and bleeding were 18.0%, 1.2%, 3.9%, 0.4%, 1.9% and 1.7%, respectively. Besides, the independent factors related to AEs, ADRs, thrombocytopenia and bleeding were recognized, which mainly included history of cardiac surgery and renal function impairment, high CRUSADE risk stratification, elective operation and combination with GP IIb/IIIa inhibitors, and so on. Moreover, 4 multivariate model was constructed based on the independent risk factors, which presented acceptable predictive values for AEs, ADRs, thrombocytopenia and bleeding.

Since its synthesis, bivalirudin has always been considered as an alternative anticoagulant. Initially being evaluated in patients receiving angioplasty, the BAT trial reveals that bivalirudin reduces the incidence of bleeding complication compared with heparin [23]. Later in patients receiving PCI, bivalirudin also presents superiority in decreasing the risk of major bleeding compared with heparin [24, 25]. Meanwhile, the HORIZONS-AMI trial reveals that bivalirudin reduces 30-day major bleeding (4.9%) compared with heparin plus GP IIb/IIIa inhibitors (8.3%) in STEMI patients undergoing PCI [26]; the EUROMAX trial finds that bivalirudin decreases mortality or major bleeding at 30 days (5.1%) compared to heparin or enoxaparin (8.5%) in STEMI patients receiving PCI [16]. However, since bivalirudin is recently applied in China, studies investigating the safety profile of bivalirudin in Chinese patients receiving PCI are still less. The BRIGHT trial enrolls 2194 patients with AMI receiving PCI from 82 centers in China and compares the 30-day net adverse clinical events of bivalirudin against heparin with or without tirofiban as anticoagulants in these patients [17], which remains the only large-scale clinical trial evaluating the safety profile of bivalirudin against heparin in Chinese patients receiving PCI. However, more studies should be conducted to assess the tolerance of bivalirudin in Chinese patients receiving PCI, serving as the basis for the administration of bivalirudin in these patients. Therefore, the present prospective, multi-center, intensive monitoring study was conducted and observed that the incidences of AEs, SAEs, ADRs and SADRs were 18.0%, 1.2%, 3.9% and 0.4%, respectively in 1224 STEMI patients undergoing PCI receiving bivalirudin as anticoagulant from 27 Chinese centers. Compared to previous clinical trials, the present study did not observe excessive AEs [17, 27, 28]; whereas the incidence of ADRs could not be referred since there existed few previous data on this. In the present study, it was also observed that the incidences of thrombocytopenia and bleeding were 1.9% and 1.7%, accordingly. The incidence of bleeding was numerically lower than previous studies [16, 17, 26], which could be explained by different clinical settings such as follow-up period and PCI detail between previous studies and the present study.

Furthermore, the present study also investigated the risk factors related to AEs, ADRs, thrombocytopenia and bleeding in Chinese STEMI patients undergoing PCI and receiving bivalirudin as anticoagulant, which identified history of cardiac surgery and renal function impairment, high CRUSADE risk stratification, elective surgery and combination with GP IIb/IIIa inhibitors. The CRUSADE risk stratification is a well-known predictive model for in-hospital major bleeding, which includes prior vascular diseases as one of its indexes [22]; this could explain our finding that history of cardiac surgery was an independent risk factor for AEs, ADRs and thrombocytopenia. Meanwhile, renal function impairment is also a well-recognized risk factor for AEs including bleeding [29], which was in line with our finding. Besides, combination of GP IIb/IIIa inhibitors was a risk factor for AEs, ADRs and thrombocytopenia, which indicated that GP IIb/IIIa inhibitors might be carefully used in STEMI patients undergoing PCI and receiving bivalirudin as anticoagulant. Apart from that, the present study also established 4 multivariate models based on the above-mentioned factors to predict the incidence of AEs, ADRs, thrombocytopenia and bleeding, which revealed all the 4 multivariate models possessed acceptable predictive value. These findings suggested that these multivariate models might be potential tools to recognize STEMI patients undergoing PCI and receiving bivalirudin as anticoagulant with high risk of AEs, ADRs, thrombocytopenia and bleeding, which could potentially improve their outcome.

Although several interesting findings were revealed, there existed limitations in the present study. Firstly, although the present study had a relatively large sample size, the incidences of ADRs including thrombocytopenia and bleeding were relatively low, which might cause insufficient statistical power. Secondly, this study was an observational study with single-arm, further randomized, controlled trials should be conducted to verify the findings of the present study.

To be conclusive, bivalirudin exhibits well-pleasing safety profile in Chinese STEMI patients undergoing PCI, reflecting by its low incidence of AEs, ADRs, thrombocytopenia and bleeding.

## Supplementary Information

**Additional file 1. Supplementary table 1.** Detailed AEs and ADRs in System Organ Class (SOC).

## Data Availability

The datasets supporting the conclusions of this article are included within the article and its additional files.
