# Supplementary material for: Comprehensive safety profile evaluation of bivalirudin in Chinese ST-segment elevation myocardial infarction patients receiving percutaneous coronary intervention: a prospective, multicenter, intensive monitoring study
Source: BMC Cardiovasc Disord. 2022 Jun 25;22:290. doi: 10.1186/s12872-022-02716-4 (PMC9233771; doi:10.1186/s12872-022-02716-4)
Supplement: Supplementary file 1 — Additional file 1. Supplementary table 1. Detailed AEs and ADRs in System Organ Class (SOC). [file 12872_2022_2716_MOESM1_ESM.docx]

**Supplementary table 1.** Detailed AEs and ADRs in System Organ Class (SOC)

| Items | AEs, No. (%) | | | | ADRs, No. (%) | | | |
| --- | --- | --- | --- | --- | --- | --- | --- | --- |
|  | Total | Mild | Moderate | Severe | Total | Mild | Moderate | Severe |
| Total | 224 (18.0) | 187 (15.0) | 23 (1.8) | 14 (1.1) | 49 (3.9) | 44 (3.5) | 4 (0.3) | 1 (0.1) |
| Gastrointestinal disorders | 70 (5.6) | 65 (5.2) | 3 (0.2) | 2 (0.2) | 12 (1.0) | 10 (0.8) | 1 (0.1) | 1 (0.1) |
| Respiratory, thoracic, and mediastinal disorders | 60 (4.8) | 57 (4.6) | 3 (0.2) | 0 (0.0) | 1 (0.1) | 1 (0.1) | 0 (0.0) | 0 (0.0) |
| General disorders and administration site conditions | 57 (4.6) | 50 (4.0) | 4 (0.3) | 3 (0.2) | 2 (0.2) | 0 (0.0) | 2 (0.2) | 0 (0.0) |
| Investigations | 43 (3.5) | 37 (3.0) | 6 (0.5) | 0 (0.0) | 4 (0.3) | 4 (0.3) | 0 (0.0) | 0 (0.0) |
| Cardiac disorders | 31 (2.5) | 23 (1.8) | 2 (0.2) | 6 (0.5) | 0 (0.0) | 0 (0.0) | 0 (0.0) | 0 (0.0) |
| Blood and lymphatic system disorders | 26 (2.1) | 25 (2.0) | 0 (0.0) | 1 (0.1) | 25 (2.0) | 25 (2.0) | 0 (0.0) | 0 (0.0) |
| Nervous system disorders | 20 (1.6) | 16 (1.3) | 2 (0.2) | 2 (0.2) | 1 (0.1) | 1 (0.1) | 0 (0.0) | 0 (0.0) |
| Renal and urinary disorders | 20 (1.6) | 19 (1.5) | 1 (0.1) | 0 (0.0) | 1 (0.1) | 1 (0.1) | 0 (0.0) | 0 (0.0) |
| Infections and infestations | 11 (0.9) | 8 (0.6) | 3 (0.2) | 0 (0.0) | 0 (0.0) | 0 (0.0) | 0 (0.0) | 0 (0.0) |
| Hepatobiliary disorders | 9 (0.7) | 8 (0.6) | 1 (0.1) | 0 (0.0) | 0 (0.0) | 0 (0.0) | 0 (0.0) | 0 (0.0) |
| Skin and subcutaneous tissue disorders | 8 (0.6) | 8 (0.6) | 0 (0.0) | 0 (0.0) | 2 (0.2) | 2 (0.2) | 0 (0.0) | 0 (0.0) |
| Musculoskeletal and connective tissue disorders | 8 (0.6) | 7 (0.6) | 1 (0.1) | 0 (0.0) | 1 (0.1) | 1 (0.1) | 0 (0.0) | 0 (0.0) |
| Psychiatric disorders | 8 (0.6) | 7 (0.6) | 0 (0.0) | 1 (0.1) | 0 (0.0) | 0 (0.0) | 0 (0.0) | 0 (0.0) |
| Metabolism and nutrition disorders | 7 (0.6) | 7 (0.6) | 0 (0.0) | 0 (0.0) | 0 (0.0) | 0 (0.0) | 0 (0.0) | 0 (0.0) |
| Vascular disorders | 7 (0.6) | 7 (0.6) | 0 (0.0) | 0 (0.0) | 0 (0.0) | 0 (0.0) | 0 (0.0) | 0 (0.0) |
| Eye disorders | 2 (0.2) | 2 (0.2) | 0 (0.0) | 0 (0.0) | 0 (0.0) | 0 (0.0) | 0 (0.0) | 0 (0.0) |
| Injury, poisoning and procedural complications | 1 (0.1) | 1 (0.1) | 0 (0.0) | 0 (0.0) | 1 (0.1) | 1 (0.1) | 0 (0.0) | 0 (0.0) |
| Endocrine disorders | 1 (0.1) | 1 (0.1) | 0 (0.0) | 0 (0.0) | 0 (0.0) | 0 (0.0) | 0 (0.0) | 0 (0.0) |
| Reproductive system and breast disorders | 1 (0.1) | 1 (0.1) | 0 (0.0) | 0 (0.0) | 0 (0.0) | 0 (0.0) | 0 (0.0) | 0 (0.0) |
| Immune system disorders | 1 (0.1) | 1 (0.1) | 0 (0.0) | 0 (0.0) | 0 (0.0) | 0 (0.0) | 0 (0.0) | 0 (0.0) |

AEs, adverse events; ADRs, adverse drug reactions.
